# Targeted Therapy for Complex Lymphatic Anomalies in Patients with Noonan Syndrome and Related Disorders

**DOI:** 10.3390/ijms26136126

**Published:** 2025-06-26

**Authors:** Erika K. S. M. Leenders, Vera C. van den Brink, Lotte E. R. Kleimeier, Danielle T. J. Woutersen, Catelijne H. Coppens, Jeroen den Hertog, Willemijn M. Klein, Tuula Rinne, Sabine L. Vrancken, Saskia N. de Wildt, Jos M. T. Draaisma, Joris Fuijkschot

**Affiliations:** 1 Department of Human Genetics, Radboud University Medical Center, 6525 GA Nijmegen, The Netherlands; 2 Donders Institute for Brain, Cognition and Behavior, Radboud University Medical Center, 6525 GA Nijmegen, The Netherlands; 3 Department of Pediatrics, Amalia Children’s Hospital, Radboud University Medical Center, 6525 GA Nijmegen, The Netherlands; 4 Radboud Institute for Health Sciences, Radboud University Medical Center, 6525 GA Nijmegen, The Netherlands; 5 Hubrecht Institute-KNAW, University Medical Center Utrecht, 3584 CT Utrecht, The Netherlands; 6 Institute Biology Leiden, Leiden University, 2333 BE Leiden, The Netherlands; 7 Department of Medical Imaging, Radboud University Medical Center, 6525 GA Nijmegen, The Netherlands; 8 Department of Perinatology (Neonatology), Amalia Children’s Hospital, Radboud University Medical Center, 6525 GA Nijmegen, The Netherlands; 9 Department of Pharmacy, Pharmacology and Toxicology, Radboud University Medical Center, 6525 GA Nijmegen, The Netherlands

**Keywords:** trametinib, Noonan syndrome, CCLA, lymphatic anomaly, RASopathies, targeted therapy, genetics

## Abstract

Recent diagnostic advances reveal that lymphatic disease in Noonan syndrome (NS) and other NS-like RASopathies often stems from central conducting lymphatic anomalies (CCLAs). The RAS/MAPK-ERK pathway plays a central role in lymphangiogenesis. Targeting this pathway with MEK-inhibitor trametinib has emerged as a promising therapeutic strategy for managing CCLAs in patients with NS-like RASopathies. This case series assessed the clinical outcomes of trametinib therapy in eight patients with NS-like RASopathies and CCLA, each offering unique insights into the therapeutic efficacy of MEK inhibition. In infants, a lower dose of 0.01 mg/kg/day and earlier discontinuation of trametinib therapy effectively alleviated the symptoms of congenital chylothorax and rescued the lymphatic phenotype, compared to similar published cases. Moreover, four patients aged >11 y showed a slower response and did not achieve complete symptomatic recovery. In conclusion, it is advised to consider trametinib therapy for patients with severe, therapy-refractory CCLA in patients with NS-like RASopathies. However, individual responses to trametinib therapy may vary, with some patients demonstrating more favorable outcomes than others. Further investigation into potential enhancers and suppressors of the lymphatic phenotype is necessary for more accurate treatment predictions. While these factors are likely genetic, we cannot rule out other intrinsic or physiological factors.

## 1. Introduction

Noonan syndrome (NS) is a primarily autosomal dominant disorder characterized by a variable developmental delay, short stature, congenital heart defects, distinctive facial features, and lymphatic dysplasia. NS is caused by germline pathogenic variants in genes that encode for key components of the RAS/mitogen-activated protein kinase (MAPK) signaling pathway, which plays a critical role in the regulation of cell differentiation, proliferation, and survival. NS is part of a larger ‘RASopathy’ family, which also includes other disorders such as Cardiofaciocutaneous syndrome (CFCS; MIM #PS115150), Noonan syndrome with multiple lentigines (NSML; MIM #PS151100), and mosaic RASopathies. RASopathies share a common molecular etiology where pathogenic variants cause RAS/MAPK pathway dysregulation, often leading to a hyperactivation of the downstream signal. To date, disease-causing variants in more than 20 genes have been associated with RASopathies [1].

Lymphatic anomalies are a major feature in NS, with a lifetime prevalence of approximately 37% [2]. Symptoms may present prenatally, with an increased nuchal translucency (NT) and persistent nuchal fold, hydrothorax, or fetal hydrops. Postnatal problems may include (uni- or bilateral) chylothorax, protein-losing enteropathy (PLE), (lower) limb lymphedema (LLL), or chylopericardium [2]. One of the most prevalent presentations in early infancy is chylothorax, which can be life-threatening and refractory to standard therapy. Due to the upcoming use of intranodal Dynamic Contrast-Enhanced MR Lymphangiography (DCMRL), it has become evident that lymphatic disease in RASopathies may be caused by a central conducting lymphatic anomaly (CCLA). CCLA is defined as anatomical abnormalities in the central lymphatic system leading to abnormal drainage and/or retrograde flow of lymphatic fluid [3].

**Figure 1:**
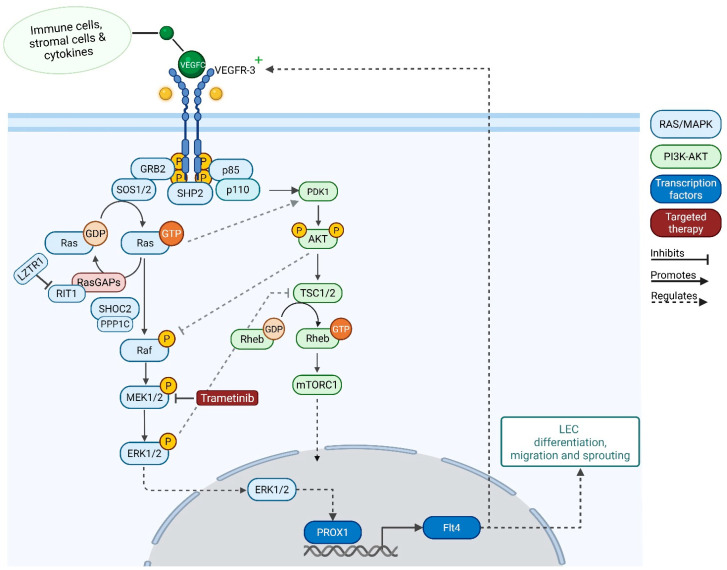
Intracellular signaling pathways in lymphatic endothelial cells (LECs) mediated through the VEGFC-VEGFR-3 ligand–receptor interaction. The activation of ERK1/2 is believed to initiate the transcription of *PROX1*, which subsequently triggers a positive feedback loop with VEGFR-3. This loop leads to the downstream activation of the RAS/MAPK pathway, resulting in ERK phosphorylation. This signaling cascade activates nuclear transcription.

The formation of the lymphatic system arises from the cardinal vein, where Vascular Endothelial Growth Factor C (VEGFC)-mediated activation of Vascular Endothelial Growth Factor Receptor-3 (VEGFR-3) activates downstream signaling cascades, resulting in the activation of MAPK-ERK and the upregulation of Prospero-related homeobox-1 (*PROX1*) in blood vascular endothelial cells (BECs), which leads to lymphatic endothelial cell (LEC) differentiation [4]. PROX1 is frequently described as the primary switch in determining LEC fate. PROX1-deficient cells fail to express lymphatic endothelial markers and subsequently retain their blood vascular endothelial phenotype, resulting in a complete or partial loss of lymphatic vessel formation [5,6]. *PROX1* regulates the transcription of numerous lymph-specific genes, with Fms-related tyrosine kinase 4 (*FLT4*) being a primary target. *FLT4* encodes for VEGFR-3 and is required for the further development of lymphatic vessels. VEGFR-3-positive LECs migrate from the cardinal vein and proliferate to form lymphatic sacs and the primitive lymphatic plexus. The main downstream effect following VEGFR-3 activation is the stimulation of the phosphoinositide-3-kinase-PKB/AKT (PI3K-PKB/AKT) and the RAS/MAPK-ERK signaling pathways (Figure 1).

There are many downstream targets of ERK, including numerous kinases and transcription factors. Excessive phosphorylated ERK enhances the expression of *SOX18* and *PROX1* and the expression of lymphatic markers, such as *FLT4*, encoding VEGFR-3. Particularly enhanced expression of *PROX1* is essential for the process of increased proliferation and outmigration of newly differentiated LECs, which results in the formation of enlarged lymphatic sacs and lymphangiectasia [4]. The group of Dellinger demonstrated that a pathogenic gain-of-function variant in *KRAS* (c.35G>A (p.Gly12Asp)) induces excessive ERK signaling, causing edema, enlarged jugular lymph sacs, and malformed lymphatic valves in mice. They also showed that hyperactive KRAS signaling in LECs leads to changes in gene expression, with numerous genes that are downregulated involved in the maturation of lymphatic vessels. Furthermore, they showed that this lymphatic phenotype could be partially ameliorated by the pharmacologic inhibition of MAPK activation using MEK-inhibitor trametinib [7,8].

When looking at the pathophysiology of lymphangiogenesis, it becomes evident that the RAS/MAPK-ERK pathway plays a key role throughout the process, from LEC fate specification to lymphatic maturation and valve formation. Trametinib is a mitogen-activated protein kinase inhibitor (MEKi) approved for the treatment of cancer caused by excessive RAS/MAPK signaling, such as metastatic melanoma and childhood glioma. The inhibition of MEK is a potential treatment for patients with NS-like RASopathies and CCLA.

The recommended trametinib dose for adult cancer treatment is 2 mg/day, equivalent to 0.025 mg/kg/day for an 80 kg adult. In children with refractory solid tumors, a phase I trial suggested doses of 0.025 mg/kg/day (<6 years) and 0.032 mg/kg/day (≥6 years) [9]. The repurposing of trametinib treatment in NS differs from its use in the treatment of cancer. Oncogenic variants are gain-of-function variants with a strong activating effect, whereas variants that cause NS tend to be germline and less activating. Also, for the treatment of NS-like RASopathies, the aim is not to induce apoptosis but rather to bring RAS/MAPK signaling down to normal levels to allow normal cellular function. Moreover, developmental changes in the processes involved in the disposition of drugs change with age [9]. As trametinib’s excretion is complicated, with multiple drug-metabolizing enzymes involved as well as the excretion in bile and urine, it is unclear how exactly age impacts trametinib clearance in neonates and young infants [10]. All these factors influence treatment strategies, dosing, and the duration of treatment. To date, 15 patients with NS and severe CCLA have been published that were treated with MEKi, with trametinib most often used in 0.025 mg/kg/day (range 0.025–0.032 mg/kg/day) [11,12,13,14,15,16,17,18,19,20,21,22,23]. In this case series, we will share our experience with trametinib treatment in eight additional patients with RASopathies and severe, therapy-refractory lymphatic disease.

## 2. Results

Eight patients with molecular confirmed NS-like RASopathy and—except for #8—radiologically diagnosed CCLA were treated with MEKi trametinib. Patients presented with a variety of symptoms related to lymphatic disease: (congenital) chylothorax (5/8), LLL (4/8) and/or scrotal leakage (1/8) and PLE (1/8). Patient age varied between 0 and 27 years. Table 1 provides an overview of the demographics, clinical characteristics, and molecular diagnosis per patient. Five of these cases have been published previously, of which four were in the context of radiological diagnoses of CCLA and one was in the context of therapy with an MEKi [3,12,22].

### 2.1. Primary Outcomes: Course of Treatment and Efficacy

An overview of the course of treatment is provided in Table 2 and Table 3. The initial dose for patients aged 11–27 years (*n* = 5) varied (0.5–1 mg once daily; 0.008–0.02 mg/kg/day). The initial dosage was increased in 4/5 patients, and treatment was temporarily discontinued due to adverse events in 2/5 patients. The mean treatment duration in this group was 450 days, ranging from 238 to 709 days. The main reason for cessation of therapy was either an adverse event with minimal or no symptom relief (4/5) or sufficient clinical improvement (1/5).

The infants (*n* = 3) received a starting dose of 0.01 mg/kg/day. Adjustments in the treatment plan were made in 2/3 patients. The total duration of treatment varied significantly (26, 85, and 321 days). The reason for cessation of therapy was a sufficient clinical response to treatment. In one case, this was combined with the need for medication that was incompatible with trametinib due to a different medical condition.

The following sections elaborate upon each category of lymphatic disease in greater detail.

#### 2.1.1. Chylothorax

Five patients from this case series were treated for (congenital) chylothorax: two patients of 16 (#5) and 20 (#4) years of age and three infants (#6–8). Prior to trametinib treatment, patients #4 and #5 had a history of recurrent therapy-refractory chylothorax over several years, despite frequent drainage interventions. In patient 4, a brief period of clinical improvement was seen after four months of treatment. This period of clinical improvement was followed by a deterioration of symptoms, including increases in weight gain and scrotal leakage. Moreover, the patient required supplementary oxygen support throughout the day. Due to the deterioration of symptoms and in consultation with the patient, it was decided to discontinue the therapy. After cessation, part of these symptoms disappeared, the scrotal leakage remained unchanged, while the swelling in the legs and abdomen decreased. At the most recent follow-up, conducted seven months after discontinuation, the symptoms of the patient were notably improved compared to before the start of trametinib. Oxygen support was only necessary at night. In patient 5, following six weeks of therapy, a single pleural drainage intervention was performed. Afterwards, the patient reported no dyspnea and ultrasounds of the chest showed no pleural effusions. No recurrence of the pleural chylous effusion was observed after, with a follow-up time of 22 months after cessation of treatment.

Three infants (#6–8) diagnosed with congenital chylothorax were treated as outlined in Table 2, Table 3 and Table 4. Chylous effusions significantly decreased within two months of treatment, allowing thoracic drains in patients 6 and 8 to be removed within a month. All patients were weaned from invasive ventilation to minimal or no respiratory support, with patients 7 and 8 achieving this within one month. Trametinib treatment was temporarily discontinued in patients 6 and 8 due to infection, with no recurrence of effusions. Propranolol was administered throughout treatment. All three infants were ultimately discharged home in good condition without trametinib.

#### 2.1.2. Lower Limb Lymphedema (LLL)

Four patients presented with lower limb lymphedema (LLL) (#1, #2, #4 #5), with a median age of 16.5 (11–20 y) years. Two received treatment for therapy-refractory primary LLL, while the other two presented with LLL secondary to chylothorax. Table 5 provides a detailed overview of visual observations during and after trametinib treatment. In all four patients, (temporary) improvements in LLL were reported, such as reduced pain and enhanced mobility. After discontinuation of trametinib for adverse events or lack of effectiveness, pain and swelling worsened in three of four patients. Compared to the visual presentation of LLL prior to the initiation of trametinib, a slight overall improvement was noted in all four cases after discontinuation.

#### 2.1.3. Protein-Losing Enteropathy (PLE)

One patient (#3) was initiated on trametinib primarily for the management of newly developed PLE, which was potentially attributable to a radiologically confirmed diagnosis of CCLA. In addition, he was diagnosed with common variable immunodeficiency (CVID) at the age of 9 years, for which he received immunoglobulin supplementation regularly. The CVID was a primary diagnosis not related to the CCLA. The patient had experienced a decline in his already low energy levels, which was accompanied by a decrease in his serum albumin level to subnormal. Despite trametinib treatment, laboratory parameters remained unchanged and the patient reported no improvement in energy levels or quality of sleep. Consequently, the dose was increased to 1.5 mg/day. Following therapy initiation, at 57 weeks, a marginal improvement in energy levels was noted. The alpha-1-antitrypsin (A1A) clearance was decreased significantly to 21.9 mL/24 h (ref < 15 mL/24 h), accompanied by the normalization of serum albumin levels. However, by week 87, the A1A clearance increased again to 110.2 mL/24 h without an identifiable cause. The patient did not report symptom relief but instead experienced a further slight decline in energy levels, leading to the discontinuation of trametinib treatment. During trametinib therapy, he suffered from acneiform eruptions managed with topical antibiotics. Notably, two years after cessation of treatment, the serum albumin levels were normal.

### 2.2. Secondary Outcomes: Adverse Events and Radiology Assessment

#### 2.2.1. Adverse Events and Deviation from Course of Treatment

In four patients (#4–6, #8), therapy was temporarily halted due to adverse events, which included blurry vision, fever, or infections. If necessary, the interruptions were managed with supportive care, and trametinib treatment was resumed once the adverse events were treated or had resolved spontaneously. In four patients (#1, #2, #4, and #7), therapy was terminated because of adverse events without any deterioration of symptoms. The results are further summarized in Table 3.

#### 2.2.2. Radiology Assessment Before and After Treatment

**Figure 2:**
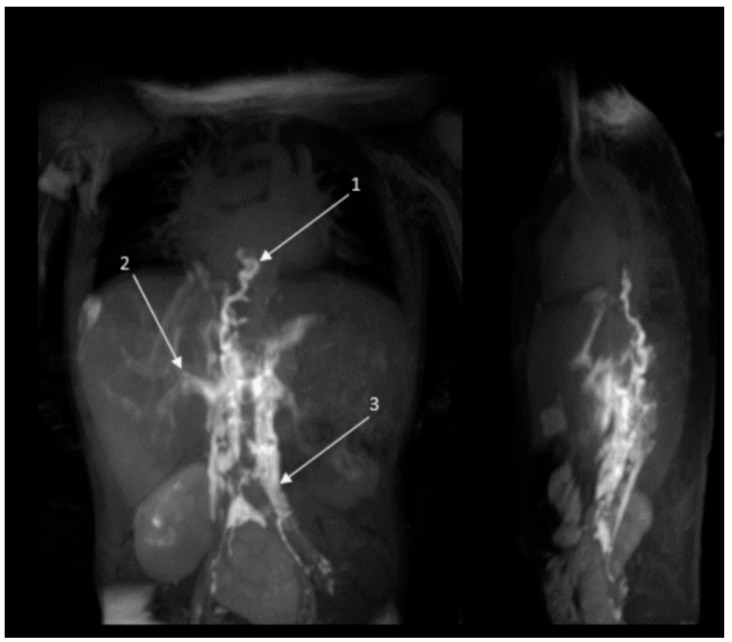
DCMRL imaging before treatment of patient #2. Before treatment, contrast did not reach beyond the diaphragm (1). Additionally, severe hepatic (2) and retroperitoneal (3) backflow was observed following intranodal injection.

Before the start of treatment, the anatomy of the lymphatic system and lymphatic flow patterns were evaluated in 7/8 patients with the use of DCMRL. In one patient (#8), no DCMRL was performed due to logistical constraints; diagnosis of CCLA was therefore based upon the combination of a congenital chylothorax and NS. Anatomical abnormalities were observed in 6/7 patients, including (partial) aplasia of the thoracic duct, lymphangiectasia, or ectatic lymphatic tracts. This is visually represented in Figure 2. Retrograde lymphatic flow, dermal backflow, or severe mesenterial, interstitial, or subcutaneous edema were observed in all patients. A full overview of the DCMRL reports of patients #1–4 and #6 was previously published [3,12].

After cessation of trametinib, the central lymphatic system in 3/8 patients was reassessed using either T2 MRI imaging (2/3) or DCMRL (1/3). The DCMRL showed a normalized slim and full-length thoracic duct (TD), whereas no contrast was visible in the thoracic duct on the initial imaging. In patient #5, T2 and #6 DCMRL images showed an overall decrease in edema and pleural fluids. In patient #1, edema and extensive lymphangiectasia were unchanged. No patients underwent surgical intervention during or following the DCMRL procedure.

Based on the DCMRL observations, seven patients were diagnosed with CCLA. The radiologically confirmed CCLA diagnosis, along with the clinical presence of severe lymphatic disease, prompted the initiation of trametinib treatment in patients between the ages of 11 and 27 years. The three infants were started on trametinib based on a combination of DCMRL findings, genetic diagnosis, clinical presentation, and the exhaustion of all other conservative treatment options.

## 3. Discussion

This case series evaluated the effectiveness of treatment with MEKi trametinib in eight patients diagnosed with NS-like RASopathies with variable presentations of lymphatic dysfunction. Our findings emphasize the variability in symptoms prompting the initiation of targeted therapy and the diverse clinical outcomes observed during and after trametinib treatment. The three infants responded faster to trametinib treatment, in comparison to the older children and adults, where the treatment effect was more variable and delayed.

### 3.1. Dosing Strategy and Treatment Effect

Current dosing strategies for NS-like RASopathies mainly rely on case reports and small series across ages and symptoms, with trametinib most often used at a dose of 0.025 mg/kg/day (range 0.025–0.032 mg/kg/day). In most published cases, including our case series, treatment was not guided by therapeutic drug monitoring as target trough levels for complex lymphatic anomalies are not known. In a recent study where two infants of three months old with Noonan syndrome and hypertrophic cardiomyopathy were treated, using a dose range of 0.02–0.04 mg/kg/day, trametinib trough levels of 4–7 ng/mL were proposed, which are lower than for oncological indications (7–17 ng/mL) [24]. In our case series, dosing ranged from 0.005 to 0.03 mg/kg/day, with treatment durations varying from 26 days to over two years, depending on the patient’s age and specific clinical requirements.

Three infants with congenital chylothorax/hydrops fetalis showed consistent clinical improvement with a lower dose, shorter treatment duration than described thus far, and a low incidence of side effects (Table 3). In contrast, patients > 1 year of age in this case series were treated with higher doses over extended periods. The first neonate that received treatment (patient 6) was treated with a higher dose (0.018 mg/kg/day) than patients 7 (0.01 mg/kg/day) and 8 (0.005–0.01 mg/kg/day). These lower doses led to a comparable treatment effect without common side effects of trametinib such as pyrexia and skin reactions. We identified nine additional trametinib-treated cases of infants with congenital (bilateral) chylothorax [13,16,17,19,20,21,22,23]. The starting dose in the published cases ranged from 0.01 to 0.032 mg/kg/day and all also demonstrated a fast clinical improvement. Based on these 12 cases and the prognosis of hydrops fetalis in infants [25], trametinib appears to be particularly effective in the treatment of congenital chylothorax, with a favorable clinical course observed in infants in this cohort, consistent with previously reported cases. Based on previous cohort studies on congenital chylothorax in general, the overall survival with supportive management (e.g., MCT diet, thoracic drainage) is estimated to be around 68% [26]. Additionally, mortality rates among patients with NS-like RASopathies and congenital chylothorax are unknown, hindering comparison. Therefore, it remains unclear whether the favorable trajectories reflect the treatment response or the potential natural course of the disease.

The treatment effect in adolescent patients treated for chylothorax varied. The reason for the lack of a durable response in patient #4 remains unclear, as this is inconsistent with previously published cases. Swelling and edema are common side effects of trametinib, which may partially explain the relapse of symptoms. Thus far, six additional patients with NS have been published with (bilateral) chylothorax that presented beyond infancy (ages between 3 and 22 years) and were treated with trametinib (Appendix A) [11,12,15,16,18,27]. In all published cases, a positive effect was seen on pulmonary function and/or the reduction of pleural effusion. Notable was the oldest patient (22 years), for whom the highest dose (2 mg/day) and longest treatment duration (28+ months) were needed.

Subjective improvements were reported in all LLL patients, but objective assessments failed to demonstrate a sustained therapeutic effect. To date, other published cases of LLL in NS-like RASopathies for which targeted treatment was initiated have not been published. Based on these four patients, our data does not support MEKi treatment in patients with NS-like RASopathies and LLL as a primary therapeutic target.

Case 3 highlights the clinical challenges associated with managing PLE in the context of multiple comorbidities. A recently published case report described a 14-year-old female with PLE and a different pathogenic variant in *SOS1* in whom MEKi therapy led to the complete resolution of symptoms and the normalization of hematological and biochemical parameters. In contrast, our patient presented with a more complex clinical profile, including severe fatigue, CVID, CCLA, and PLE. Fatigue is a well-recognized symptom in patients with CVID, and PLE can develop as a complication of both CVID and CCLA [28,29]. Interestingly, the patient’s serum albumin levels normalized two years after discontinuing therapy, without additional interventions. It remains unknown why the patient improved without therapy. However, this raises the question of whether PLE in this case primarily resulted from CVID, possibly exacerbated by the presence of CCLA.

### 3.2. Potential Mechanisms Underlying Therapeutic Response

A possible explanation for the remarkably fast improvement in infants, in comparison to the pediatric and adolescent patients, could be that there is a ‘window of opportunity’ in the early postnatal phase during which the lymphatic system is still maturing and is therefore more susceptible to modulation. During the first months after birth, the lymphatic system still undergoes critical developmental changes, such as vessel maturation, the formation and maturation of lymphatic valves, and the transition of LEC connections from zipper-like to button-like junctions [30]. Also, during the first weeks after birth, the VEGF-C/VEGFR-3 pathway is still essential for maintaining lymphatic vasculature. Beyond this period, the survival of lymphatic vessels becomes independent of VEGFR-3 ligands, indicating that the lymphatic capillary maturation occurs in these early weeks of life [31]. This theory is supported by evidence that trametinib treatment increases the expression of several genes that promote the maturation of lymphatic vessels [8].

Another factor that could have contributed to the clinical improvement in the three infants is that propranolol was co-administered with trametinib. Based on the clinical course, it is believed that propranolol may have contributed to the overall positive therapeutic outcome, with a lower MEKi dosing regimen than has been thus far described and a shorter duration of trametinib treatment. Propranolol is a non-selective beta-adrenergic receptor blocker, which is commonly used for the treatment of hypertension and infantile hemangiomas. It has anti-proliferative, anti-migratory, and anti-angiogenic properties and has previously been studied in various diseases, such as lymphatic disease and cancer [32,33]. The precise mechanism of action of propranolol in lymphatic disease remains to be determined, but studies suggest that propranolol exerts its effect through the reduction of VEGF expression and by reducing circulating pro-lymphangiogenic factors such as VEGF-C [34]. There is also evidence that propranolol inhibits proliferation by directly inhibiting the phosphorylation of AKT and MAPK pathways [35]. The potential synergistic effect of this dual approach warrants further investigation.

### 3.3. Future Research Directions

Off-label use of (targeted) treatment is common, particularly in the pediatric population, where many drugs are prescribed off-label [36]. However, this often leads to insufficient dosing information, increasing the risk of suboptimal treatment and adverse drug reactions. To date, no clinical trials have been initiated for this population, but such studies are crucial to establish clear treatment protocols for both pediatric and adult patients across various indications. Although therapeutic drug monitoring (TDM) was not performed in this case series, future studies should incorporate pharmacokinetics to define target trough levels for different subgroups.

Just as for targeted treatment in cancer, we believe that trametinib will not be effective in all patients and that it will depend at least on the underlying pathogenic variant, its functional impact on various pathways, the cellular context and patient characteristics, such as age. Other compounds should also be explored to further personalize treatment strategies for this genetically heterogeneous population.

As trametinib influences numerous downstream targets and pathways by the up- and downregulation of genes, co-administration with other drugs could be explored. Trametinib increases AKT phosphorylation, which is also a known mechanism involved in MEKi-related resistance in cancer treatment [37]. The secondary upregulation of interconnected pathways may impact treatment efficacy. Recently, the potential efficacy of combining trametinib with mTOR inhibitor sirolimus was evaluated as a treatment regimen for a patient with a highly symptomatic complex lymphatic anomaly (CLA), refractory to monotherapy of either drug [38].

To advance our understanding of the pathophysiology underlying lymphatic anomalies in NS-like RASopathies, future studies utilizing zebrafish models could provide valuable mechanistic insights. Previous work has shown that the expression of activating *KRAS* variants in zebrafish mimics key features of human lymphatic disease, including vessel dysplasia and edema. Notably, these models also demonstrate therapeutic responsiveness to MEK inhibition, highlighting their potential for preclinical research [39].

Radiological imaging could be used to monitor lymphatic system remodeling to potentially validate therapeutic effects. However, due to the invasive nature of the DCMRL, a follow-up DCMRL was not performed in six out of seven cases. Less invasive methods to monitor lymphatic flow recovery could be explored. High-resolution ultrasonography presents a promising alternative, potentially eliminating the need for full anaesthesia in neonates and young children, reducing procedure time, and offering a more accessible option for hospitals that cannot facilitate DCMRL.

### 3.4. Strengths and Limitations

This report has several limitations, including the small sample size, the retrospective nature of our study and the heterogeneity of the patient population, which ranged from neonates to young adults. These factors limit the generalizability of the results, as outcome measures were not standardized. Additionally, the short-term follow-up in the congenital chylothorax cases restricts the assessment of long-term outcomes in terms of safety and efficacy. Moreover, many cases of congenital chylothorax will resolve naturally over time with supportive management. The effect of trametinib is therefore difficult to determine. More studies are needed on the natural history of congenital chylothorax in NS-like RASopathies specifically.

As trametinib trough levels were not measured in our patients, non-adherence cannot be excluded. However, given the limited data, no conclusions can be drawn regarding the effectiveness of MEKi, highlighting the need for further investigation.

## 4. Methods and Materials

This case series investigated the clinical presentation, diagnostics, (therapeutic) management, and clinical outcomes of patients with NS-like RASopathies and CCLA treated with trametinib in the Radboudumc University Medical Center (Nijmegen, The Netherlands) between January 2021 and August 2024. Included patients received trametinib treatment based on the reported functional evidence demonstrating that the germline mutation led to the upregulation of the RAS-MAPK pathway or, in cases where no direct functional evidence was available, predicted RAS-MAPK hyperactivation. Patients were not routinely tested for additional somatic variants associated with their lymphatic phenotype. Patients were identified through a retrospective search of electronic medical records. The Medical Ethics Committee at Radboud University Medical Center Nijmegen granted a waiver for this study, according to the Dutch Law on Human Research (case numbers 2024-17537 and 2023-16807). Informed consent for the scientific use of medical data was obtained from patients and/or legal representatives.

The decision to start treatment was made by a multidisciplinary team with specific expertise in RASopathies and/or on the use of targeted pathway modulating treatment. Prior to treatment initiation, routine chemistry, hematology, and coagulation labs were checked. Additionally, an ophthalmological examination, electrocardiogram (ECG), and echocardiography were performed to identify pre-existing conditions that could increase the risk of adverse events. Patients received trametinib as Mekinist^®^ (tablet) or Spexotras^®^ (liquid suspension). Treatment was initiated as off-label use. The liquid suspension (Spexotras^®^) was provided through the Novartis (Basel, Switserland) compassionate use program for neonates and patients who could not swallow tablets or required weight-based dosing. As Spexotras^®^ was not on the Dutch market prior to January 2024, for these cases, formal approval was obtained from the Dutch Health Care and Youth inspectorate.

Patient demographics, clinical presentation, diagnostic findings, radiology reports, course of treatment, and clinical outcomes were extracted from the electronic medical records and entered into an electronic Case Report Form (Castor EDC, Amsterdam, The Netherlands).

### Outcome Assessment

Outcome measures varied between patients due to differing clinical presentations of CCLA and treatment-related measurements. Primary outcomes comprised the course of treatment and treatment efficacy. The course of treatment included the duration, dosing regimen, and alterations in the regime. Treatment efficacy was identified based on each patient’s lymphatic phenotype. For patients presenting with chylothorax, outcome measures included chylous drainage output, respiratory status, and radiological assessment. In patients with PLE, A1A clearance and serum albumin levels were used. To evaluate the severity of lower limb and genital lymphedema, doctors’ reports on physical examinations and patient-reported outcomes (e.g., pain, edema, energy levels, and mobility) using the ten-point Visual Analogue Score (VAS) for pain and quality of life were used.

Secondary outcome measures included the presence of adverse events, deviations in the course of treatment, and DCMRL imaging of the lymphatic system. The presence of adverse events was routinely assessed through chemistry, hematology, and coagulation labs, as well as ophthalmologic examinations. Adverse events leading to (temporary) treatment discontinuation were documented. The initial radiological diagnosis was based on DCMRL imaging and performed prior to the start of treatment due to the clinical suspicion of apparent abnormal central lymphatic flow. Gadolinium-based contrast was injected into the inguinal lymph nodes and its propagation was assessed using T1-weighted MR sequences. MR images were analyzed and reported using a standardized scoring system, as described by Kleimeier et al. [3]. Further radiological assessment included chest X-rays and thoracic ultrasounds to evaluate pleural effusions.

## 5. Conclusions

Targeted treatment of lymphatic disease in NS-like RASopathies remains challenging, as evidence suggests that multiple pathways can contribute to disease pathophysiology. A precise understanding of these underlying molecular mechanisms is essential for developing effective targeted therapies.

MEKi holds promise in managing life-threatening complications of CCLA in NS-like RASopathies, particularly in infants, where low doses and shorter treatment regimens have been associated with rapid recovery. However, research is needed to establish standardized treatment protocols tailored for various subgroups, advancing to a more personalized approach. Additionally, treatment efficacy, optimal dosing, and treatment duration likely (co-)depend on several factors such as age, clinical presentation, and underlying pathogenic variant, highlighting the need for individualized therapeutic strategies.

## Figures and Tables

**Table 1:** Demographic, clinical, and genetic features of individuals treated with trametinib.

Patient ID	1	2	3	4
Gender	F	F	M	M
Age at start of treatment (y)	17	11	27	20
Gene	*SOS2*	*SOS2*	*SOS1*	*RIT1*
Variant	c.800T>A p.(Met267Lys)	c.800T>A p.(Met267Lys)	c.1277A>C p.(Gln426Pro)	c.280G>A p.(Ala77Thr)
Clinical diagnosis	Noonan Syndrome	Noonan Syndrome	Noonan Syndrome	Noonan Syndrome
Prenatal anomalies	NE	PH	NA	NA
Cardiac anomalies	ASD, PS	PS	-	ASD, AS, PS
Primary indication MEKi	LLL	LLL	PLE	CT
Secondary indication MEKi				LLL, SL, ASC
**Patient ID**	**5**	**6**	**7**	**8**
Gender	M	M	F	M
Age at start of treatment (y)	16	0	0	0
Gene	*KRAS*	*PTPN11*	*RIT1*	*PTPN11*
Variant	c.178G>C p.(Gly60Arg)	c.922A>C p.(Asn308Asp)	c.229G>C p.(Ala77Pro)	c.317A>C p.(Asp106Ala)
Clinical diagnosis	CFC Syndrome	Noonan Syndrome	Noonan syndrome	Noonan Syndrome
Prenatal anomalies	PH, HF	HF	PH, PE, HF, UPL, PS, VSD	PH, PE, HN
Cardiac anomalies	HCM, MVR, TR, ATE	-	PST, HCM	
Primary indication MEKi	CT	CT, HF	CT	CT
Secondary indication MEKi	ASC, LLL		ASC	

AS: aortic stenosis. ASC: ascites. ASD: atrial septal defect. ATE: atrial enlargement. CT: chylothorax. HCM: hypertrophic cardiomyopathy. HF: hydrops fetalis. HN: hydronephrosis. LLL: lower limb lymphedema. MVR: mitral valve regurgitation. NA: not applicable or no data. NE: nuchal edema. PE: pleural effusion. PH: polyhydramnios. PLE: protein-losing enteropathy. PS: pulmonary (valve) stenosis. TR: tricuspid valve regurgitation. UPL: unilateral pyelectasis. VSD: ventricular septal defect.

**Table 2:** Course of treatment—trametinib.

ID	Age at Start of Treatment	Dosing Information			Total Duration of Treatment (Days)	Concurrent Treatment During Trametinib
		Starting Dose (mg/kg/day)	Days to Change Dosage	Dose After Change (mg/kg/day)		
**1**	17 Y	0.02 (1 mg/day)	-	-	335	Compression stocking
**2**	11 Y	0.018 (0.5 mg/day)	159	0.036 (1.0 mg/day)	709	Compression stocking
**3**	27 Y	0.008 (0.5 mg/day)	1st: 14 2nd: 235	1st 0.016 (1 mg/day) 2nd 0.024 (1.5 mg/day)	677	MCT
**4**	20 Y	0.0187 (1 mg/day)	118	0.028 (1.5 mg/day)	238	
**5**	16 Y	0.0139 (0.5 mg/d)	14	0.0278 (1 mg/day)	288	MCT, pleural drainage 47 days after start of trametinib
**6**	DOL29	0.0089	7	0.0178	321	Propranolol was started at a dose of 1 mg/kg/d DOL27 and discontinued after 2 weeks
**7**	DOL25	0.01	-	-	26	Propranolol was started 18 days before trametinib and continued for three months thereafter
**8**	DOL18	0.01	15 days	0.01 mg/kg/48 h	85	Propranolol was started nine days before trametinib and discontinued two months after stopping trametinib

Y: years. DOL: day of life. MCT: medium-chain triglycerides.

**Table 3:** Course of treatment—adverse events, deviations, and termination.

ID	Deviation from Course of Treatment			Finalizing Course of Treatment
	Days to Pause (Days)	Duration Pause (Days)	Adverse Events	Reason for Treatment Termination
**1**	-	-	-	Conjunctival hemorrhage
**2**	-	-	-	Fever due to cellulitis secondary to unguis incarnatus
**3**	-	-	-	No alleviation of symptoms
**4**	90	4	Blurry vision (refractive error), weight gain, and abdominal swelling	Increased abdominal swelling, persistent scrotal leakage, low hemoglobin levels
**5**	42	5	Fever and elevated CRP	No pleural effusions on ultrasound or CT scan, no further improvement of LLL
**6**	14	7	Urosepsis	Stable condition and fever
**7**	-	-	-	Stable condition and necessity to treat congenital CMV with valganciclovir (drug interaction)
**8**	3	12	Infection of thoracic drain, restart on alternate day regimen due to stable condition	Symptom-free, stable condition

CMV: cytomegalovirus. CRP: C-reactive protein. LLL: lower limb lymphedema.

**Table 4:** Clinical effect measures congenital chylothorax.

ID	Start Therapy (DOL)	Clinical Effect Measures				
		Removed Chest Tube		Wean off Positive Pressure Ventilation	Wean off Oxygen Support	Other Observations During Trametinib
		Right Side	Left Side			
**6**	27	DOL50 (spontaneous dislocation)	DOL65	DOL69 extubationDOL72 CPAP	Variable high-flow—low-flow oxygen support until DOL150	
**7**	25	NA	NA	DOL29 extubation, DOL31 CPAP to high flow	DOL39 high flow to low flow, discharged home on minimal oxygen supplementation (0.1 L 100%)	Hypertension resolved spontaneously after discontinuation
**8**	18	DOL27	DOL22	DOL25 extubation to CPAP, DOL41 CPAP to high flow, DOL56 high flow to low flow	DOL60	

DOL: day of life. NA: not applicable.

**Table 5:** Clinical effect measures for lower limb lymphedema.

ID	Age at Start of Treatment	Primary or Secondary LLL	Observations During Treatment		After Treatment
			First Improvements	Recurrence of Symptoms	
**1**	17 Y	Primary	After 43 days, ambulation for longer distances, decreased pain levels (4.5/10), patient reported improved QoL (8.5/10).	26 weeks after treatment initiation: patellar luxation and exacerbation of lymphedema in that limb. No recurrence of pain.	48 weeks after discontinuation, recurrence of pain, with an average intensity score of 2–3/10. Edema was present but appeared less pronounced compared to its presentation at the initiation of trametinib therapy.
**2**	11 Y	Primary	After 131 days, continuous improvement with only mild residual edema in feet and ankles. Pain levels decreased, reaching a score of 4/10, 57 weeks after initiation of treatment.	After 71 weeks, the patient experienced a recurrence of swelling in the feet, coinciding with warmer weather, accompanied by exacerbated pain.	6 weeks after discontinuation, no edema on the left side, but significant edema on the right. The edema had worsened since previous evaluations.
**4**	20 Y	Secondary	After 4 months, able to ambulate, scrotal leakage and swelling decreased.	Therapy was discontinued due to increased abdominal swelling; persistent leakage from the arms, legs and scrotum; and low hemoglobin.	
**5**	16 Y	Secondary	At physical examination, LLL appeared less voluminous even in the absence of compression stocking.		20 months after discontinuation, the LLL was comparable to the time before trametinib treatment.

Y: years. LLL: lower limb lymphedema.

## Data Availability

The data used in this study were obtained from patient medical records from the Radboud University Medical Center and are not publicly available due to privacy and ethical restrictions. Access to the data may be granted upon reasonable request and with appropriate ethical approvals from the institutional review board.

## Appendix A

**Table A1:** Overview of the literature.

Author	M/F	Affected Gene	Age at Start of Therapy	Initial Diagnosis	Starting dose (mg/kg/d)	Protocol Alterations	Duration Treatment	Clinical Effect Measures			
								Removed Chest Tube	Wean off Positive Pressure Ventilation (Weeks)	Wean off Oxygen Support	Other
Li (2019) [12]	M	*ARAF*	12 y	CCLA	1 mg/d	NR	NR	NR	NR	NR	Improvement in pulmonary function within 2 months of therapy. After 12 months of therapy, FEV1 improved from 23% to 42%. Electrolytes normalized.
Andelfinger (2019) [23]	F	*RIT1*	13 w	CT, HCM	0.02	-	17 m	6 w	NR	NR	In addition to propranolol (10 mg/kg/d), chylothorax resolved 33 days after initiation of treatment. After 17 months follow-up, stable at home.
Dori (2020) [11]	F	*SOS1*	14 y	new-onset PLE, left CT	0.01	Increased to 0.02 within 1 week	6 m+	NA	NA	NA	Albumin and hemoglobin levels normalized and no longer needed supplementation or transfusions. Electrolytes also normalized.
Hofbeck (2021) [18]	F	*PTPN11*	7 y	Post surgical CT, LLL	0.01	After 1 week, increased to 0.02	9 m	NR	NR	NR	CT resolved, lymphedema improved. After 9 months follow-up, stable at home
Meisner (2021) [21]	F	*RAF1*	20 w	CT, HCM, MAT	0.025	Stop for 3 days due to side effects; restarted at 0.01875	NR	NR	NR	NR	Propranolol, CT resolved, stable after 9 months follow-up
Gordon (2022) [27]	M	*RIT1*	22 y	CT, LLL, GL	1 mg/d	Increased to 2 mg/d	Ongoing (28+ m)	-	-	-	Albumin levels improved and resolution of ascites within 3 months. Pericardial effusions resolved after 18 months. Improvement in QoL. Increased oxygen saturations. No pleural effusions.
Nakano (2022) [16]	F	*SOS1*	3 m	Bilateral CT and ascites	0.026	Dose was not increased with weight gain; dose at termination of therapy 0.013	12 m	4 w	4 w	5 m	Serum albumin improved dramatically over the first 9.5 months. No recurrence of chylous effusions in first 6 months after discontinuation.
Nakano (2022) [16]	M	*PTPN11*	4 m	CT, PL, and HCM	0.023	-	-	4 w	4 w	NA	The patient was discharged to outpatient management. Deceased several weeks after being discharged due to suspected sudden cardiac event.
Nakano (2022) [16]	F	*RIT1* and VUS in *PTPN11*	4 y	Bilateral CT	0.018	-	24 m	4 w	4 w	7 w	Proceeded to continue trametinib after publication, maintaining the original dose. Serum albumin improved over the first 12 months of therapy.
Lioncino (2022) [13]	M	*SOS1*	9 w	CT, PL, and MAT	0.02	-	NR		10 w	12 w	After 4 months, the patient was in good clinical condition and no recurrence of MAT.
Hribernik (2023) [15]	F	*RIT1*	3 y 4 m	Bilateral CT and HCM	0.032		3 m	NA	NA	NA	Free of CT recurrence for eight months after completing treatment.
de Brouchoven (2025) [17]	M	*PTPN11*	5 w	CT, PL, and LLL	0.010	Increased towards 0.025 mg/kg/d	5 w	2 w	NR	NR	HCM and PVS but not as indication for treatment. CT resolved, CPL improved, and PVS improved as well. Stopped due to side effect of GI bleeding. After 10 months follow-up, patient is at home with oxygen therapy.
Taliercio (2024) [19]	F	*SOS1*	3 m	CT	0.025	-	17 days	-	-	-	Persistence CT and CA; thus, comfort care was initiated and passed away 5 days after.
Pascarella (2024) [22]	F	*PTPN11*	4 m	CT and HCM	0.025	-	NR	NR	NR	NR	CT resolved and HCM improved. After 18 months follow-up, stable at home.
Torok (2024) [20]	F	*SOS1*	4 m	CT, CA, and HCM	0.02		14 m	1 w	4 w	8 w	CT and ascites resolved and HCM improved. After 14 months follow-up, stable at home.

CA: chylous ascites. CCLA: central conducting lymphatic anomaly. GI: gastrointestinal. CT: chylothorax. MAT: multifocal atrial tachycardia. NA: not applicable. NR: not reported. M: male. m: months. PL: pulmonary lymphangiectasia. VUS: variant of uncertain significance. w: weeks. y: years.
